# Net zero emission goals demand worldwide cooperation

**DOI:** 10.1093/nsr/nwad223

**Published:** 2023-08-21

**Authors:** Shi Xue Dou, Wei-Hong Lai, Chris Cook

**Affiliations:** Institute of Energy Materials Science, University of Shanghai for Science and Technology, China; Institute for Superconducting and Electronic Materials, University of Wollongong, Australia; Institute for Superconducting and Electronic Materials, University of Wollongong, Australia; Faculty of Engineering and Information Sciences, University of Wollongong, Australia

## Abstract

This perspective emphasizes the need for global cooperation and decisive action to address the urgent threat of climate change and achieve the net zero emissions goal before 2050.

The serious threat of climate change has become a reality of daily life all over the world. There is no priority facing humans at present more urgent than climate change. The goal of achieving net zero emissions (NZEs) within a time frame short enough to avoid catastrophic changes can only be accomplished through worldwide cooperation. This cooperation must be exceptionally strong. Even a weak level of cooperation will result in a delay of 40 years, which could be potentially too late to save the planet. This essay argues that China is crucial in achieving net zero, and that it is imperative for all political leaders to demonstrate vision and wisdom to urgently find a way, despite political and other differences, to unite all nations. The purpose of this unity is to focus their efforts and strengths on overcoming climate change, thus ensuring the habitability of our planet for all.

Climate change poses a threat to all countries, regardless of their political, social and economic systems. The effects of climate change are already observed in both northern and southern hemispheres, such as record-breaking heat waves, rising flood levels, unprecedented bush fire events, rapid erosion of coastlines and ocean rise affecting many coastal and island communities. Climate change is rapidly making our planet less habitable much sooner than most predicted, and the window of time available to limit climate change is closing.

It is a matter of urgency that the world take concrete action to battle climate change in a very limited time frame. We believe that close involvement and interaction with China is vital to limit temperature rise to 1.5 degrees centigrade and achieve NZE goals. As indicated by the examples given in Fig. 1, this is because the sheer volume of goods required worldwide to effectively mitigate climate change can only be accomplished by harnessing and collaborating with China's immense manufacturing, and research and development (R&D) capabilities.

**Figure 1.**
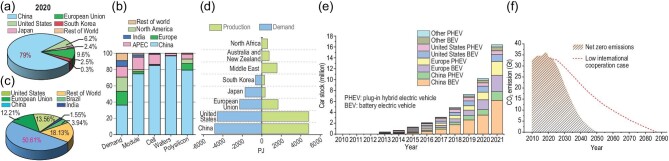
(a) The lithium-ion battery cell manufacture capacity in the world [1]. (b) Solar photovoltaics (PV) manufacturing capacity by country and region, 2021 [2]. (c) Added wind power capacity in the world in 2021 [3]. (d) Hydrogen and hydrogen-based fuel demands and production in the world by 2050 [4]. (e) The production distribution of electric cars [5]. (f) CO_2_ emissions in the low-international-cooperation case and the NZEs-goal case [6].

According to the Global Market analysis, China accounted for 79% of the world's lithium-ion battery production capacity in 2021 (Fig. 1a) [1]. It is not possible to imagine how we can achieve electrification of vehicles on the scale necessary without power sources from China. Even Tesla batteries are currently largely produced in Shanghai.

According to the International Energy Agency (IEA) prediction, meeting the goal of clean energy accounting for over 95% of the total energy supply by 2050 will require significant advancements in solar cells and wind turbines. These two sources are expected to contribute 70% of the total clean energy supply. China produced 85% of solar cells, 75% of solar cell modules and 95% of the world's wafers in 2021 (Fig. 1b) [2]. China contributed 51% of the added wind turbine capacity to the world in 2021, as shown in Fig. 1c [3].

For the world to achieve NZEs with more than 95% clean energy supply, energy storage capacity will have to increase more than an order of magnitude from the current capacity by 2050. It is evident that China will be the major contributor for battery storage supply as China is not only a major producer for all types of rechargeable batteries but also has developed supply chains for all associated raw materials and all precursor products. Although in principle some countries could over time replicate some of China's production capacities, China has taken several decades to build up the required complete production chains and this is unlikely to be achievable by other countries in the time available to avoid catastrophic climate change.

Hydrogen energy and the hydrogen economy will also play a significant role in any strategy towards achieving NZEs. This is because hydrogen, produced from renewable sources, serves as a clean energy source for fuel cells used in transportation and other applications. As shown in Fig. 1d [4], it is estimated that China will contribute ∼30% of the total hydrogen demand and production capacity by 2050. Even though this figure inevitably cannot be precisely predicted, China is also likely to have a leading position in hydrogen energy development for the foreseeable future.

It has been well established that the electric vehicle is an essential element for achieving the NZEs goal. Electric vehicle sales will have to increase 20-fold by 2040 worldwide according to a Department of Energy (DOE) report [7]. Recently reported by the IEA in its Electric Vehicle Initiative, China dominates the entire electric vehicle production stream including 50% of all electric cars (Fig. 1e) [5].

There are many more areas that demonstrate a similar scale of China's capacity in the key technologies required. For example, China provides 60% of the world's rare earth production capacity, 52% of its steel production capacity and many other important materials key to any worldwide approach to achieving NZEs.

The five elements: solar cells, wind turbines, hydrogen, energy storage and EV, are critical and decisive factors in achieving the world's NZEs goal. We believe that there is no alternative but to work closely with China in all these key areas to achieve the required NZEs outcomes within the limited time frame that is available.

The world has never been so strongly interdependent between nations and across borders. For example, lithium used in China's battery production mainly comes from Australia and Chile. Australia also supplies nearly 80% of the iron ore for the steel industry in China. Many more examples of this type demonstrate that the highly interdependent world has no choice but to work together for the common good. Even low-level cooperation will delay the achievement of the NZEs goal by as long as three to four decades, as demonstrated by the IEA in its special report [6] on Net Zero 2050, as shown in Fig. 1f. Our world cannot afford such a disastrous delay.

We acknowledge that there are also security and many other concerns to be considered when dealing with other nations. However, there is no priority facing humans at present that is more urgent than climate change. Hence, we appeal to all political leaders with vision and wisdom to urgently find a way, despite political and other differences, to unite all nations to concentrate all their efforts and strengths on overcoming climate change to ensure our planet remains habitable for all.

We suggest that the following key actions are needed:

(i) All countries should have the goal of NZEs as their highest priority in order to remove barriers to achieving the global collaborations necessary for NZEs in the limited time available.
(ii) All countries should implement free trade rules for all key productions, critical resources and supply chain materials related to NZEs in order to maximize the world's production efficiencies in manufacturing and installing equipment and systems supporting NZEs.
(iii) Support the setting up of expert international panels to identify and prioritize the critical technologies and R&D activities vital to the rapid achievement of NZEs.
(iv) Promote international cooperation and collaborative projects to enable worldwide manufacturing and R&D activities identified by the expert panels so that actions are taken with sufficient speed and on a large enough scale to ensure timely achievement of NZEs.
